# Antimicrobial use related problems and determinants in surgical ward of Ethiopian tertiary hospital

**DOI:** 10.1371/journal.pone.0296284

**Published:** 2023-12-22

**Authors:** Yirga Legesse Niriayo, Melisew Ayalneh, Gebre Teklemariam Demoz, Nigusse Tesfay, Kidu Gidey

**Affiliations:** 1 Department of Clinical Pharmacy, School of Pharmacy, College of Health Sciences, Mekelle University, Mekelle, Tigray, Ethiopia; 2 Clinical Pharmacy and Pharmacy Practice Unit, Departments of Pharmacy, College of Health Sciences, Aksum University, Axum, Tigray, Ethiopia; 3 School of dental medicine, College of Health Sciences, Mekelle University, Mekelle, Tigray, Ethiopia; International Medical University, MALAYSIA

## Abstract

**Background:**

Antibiotic use related problems lead to the emergence of resistance, failure of therapy, morbidity, mortality, and unnecessary healthcare expenditure. However, little is known about antimicrobial use related problems in our setting particularly in hospitalized surgical patients.

**Objective:**

The purpose of this study was to investigate antibiotic use related problems and their determinants among hospitalized surgical patients.

**Methodology:**

A prospective observational study was conducted from December 2018 to April 2019 at the surgical ward of the Ayder comprehensive specialized hospital, located in Northern Ethiopia. We included patients admitted to the surgical ward who were on antibiotic therapy or were candidates for antibiotic therapy/prophylaxis. The patients were recruited during admission and were followed daily until discharge. Data were collected through patient interviews and expert reviews of medical and medication records. The appropriateness of antibiotic use was evaluated according to the Infectious Disease Society of America, American Society of Health System Pharmacists, and World Health Organization guidelines. Subsequently, antibiotic use related problems were identified and classified based on Cipolle’s method followed by consensus review with experts. Binary logistic regression was performed to identify the determinants of antibiotic use related problems. Statistical significance was set at p <0.05.

**Results:**

Among 272 patients, 167(61.4%) experienced antibiotics use related problems. A total of 235 antibiotics use related problems were identified equating 0.86±0.82 problems per patient. The commonly identified antibiotic use related problems were the need for additional drug therapy (29.4%), unnecessary drug therapy (15%), and dosage too high (12.1%). Cephalosporin (47.02%) was the most commonly implicated class of antibiotics in these drug related problems, followed by penicillin (18.45%) and metronidazole (16.02%). Prolonged hospitalization (AOR: 3.57, 95% CI: 1.91–6.70), number of medications≥5 (AOR: 2.08, 95%CI: 1.10–3.94), and lower qualifications of practitioners [general practitioners (AOR: 10.27, 95%CI: 4.13–25.58) and surgical residents (AOR: 2.28, 95%CI: 1.12–4.63)] were predictors of antibiotic use related problems.

**Conclusion:**

Antibiotic use related problems were common among the hospitalized surgical patients. Prolonged hospitalizations, number of medications, and lower qualifications of practitioners were predictors of antibiotic use related problems. Therefore, more emphasis should be given for patients with prolonged hospitalization and multiple medications. Moreover, practitioners with higher qualifications including surgical specialists need to be involved in patient evaluations.

## Introduction

Ethiopia has contemplated different health financing reforms to improve the achievements of the health system goals [1]. The health care service in Ethiopia is organized into a three-tier system. These include primary, secondary, and tertiary levels of care [2]. The primary level of health care consists of primary hospitals, health centers and health posts [2, 3]. The secondary level of health care includes general hospitals that serve 1 to 1.5 million people while the tertiary level of care consists of specialized hospitals that serves 3.5 to 5.0 million people [2]. Primary health care that emphasized disease prevention and control has been the core of the country’s health system since 1993 [3]. Despite the implementation of the different health care systems, access to modern healthcare is still very limited in Ethiopia [3, 4]. Moreover, poor awareness of modern health care, shortage of medicines and equipment and lack of qualified health care personnel has been persistent problems in the Ethiopia [3, 4].

Antibiotics are antimicrobial substances that are used to treat bacterial infections by inhibiting the growth of bacteria or killing them [5]. In developing countries including Ethiopia, antibiotics are the most frequently prescribed drugs [6–8]. The use of antibiotics has been increasing worldwide particularly in developing countries [6, 9]. More importantly, antibiotics are the most frequently used drugs in surgical patients as approximately 30% of patients undergoing surgery will develop post-operative surgical site infections [10].

Antibiotics are used as prophylaxis and/ or therapeutic agents in surgical patients [11]. Although antibiotics play a crucial role in combating infectious diseases, their benefits are currently facing a great challenge due to the emergence of antibiotic resistance [12, 13]. In the 21^st^ century, antimicrobial resistance has become a serious global public health problem that threatens the effective prevention and treatment of an ever-increasing range of infectious diseases [14]. More importantly, there is a dearth of novel agents to overcome the challenge posed by resistant strains [12, 14].

Antimicrobial related problems can be classified in to seven major categories including unnecessary antimicrobial therapy, the need for additional antimicrobial therapy, ineffective antimicrobial therapy, dosage too low, dosage to high, adverse drug reaction, and noncompliance [15, 16]. Appropriate use of antibiotics is very important to prevent and control infections in surgical patients [17, 18]. However, 40–80% of the antibiotics are used inappropriately [10, 17, 19]. Globally, antibiotic use related problems are a significant public health concern and create an economic burden [20, 21]. Although antibiotic use related problems are global problems [21], they are more prevalent in developing countries including Ethiopia owing to the limited healthcare resources, poor healthcare system, and inadequately trained health care professionals in these settings [20, 22, 23]. In Sub-Saharan African countries including Ethiopia, majority of the patients (69–87%) experienced antimicrobial related problems [16, 23, 24]. Antibiotic use related problems can lead to the emergence of resistance, failure of therapy, morbidity, mortality, and unnecessary healthcare expenditure [23]. Antibiotic related problems are attributed to different factors including the reluctance of clinicians to implement clinical practice guideline recommendations, absence of well-defined protocols, complexity of the regimens, duration of hospitalization, qualification of medical practitioners, comorbidity, and polypharmacy [16, 23, 25, 26].

Globally, inappropriate use of antibiotics is a major contributing factor to the emergence and spread of antimicrobial resistance [27]. If antibiotic use related problems are not addressed in a timely manner, many of the antibiotics will be out of use due to the emergence of resistance, which in turn leads to uncontrolled bacterial infection especially in developing countries, where access to alternative medication is limited [28, 29]. Therefore, assessing the appropriateness of antibiotics use is the initial step in tackling this problem. However, little information is available regarding antimicrobial use related problems in hospitalized surgical patients in our setting. Hence, the purpose of this study was to investigate antibiotic use related problems and their determinants among hospitalized surgical patients.

## Material and methods

### Study design and study setting

A prospective observational study was conducted from December 2018 to April 2019 at the surgical ward of Ayder Comprehensive Specialized Hospital (ACSH), located in Tigray, Ethiopia. ACSH is the largest hospital in Tigray region and the second largest hospital in Ethiopia. It is a teaching and tertiary hospital serving over 10 million people in the catchment area including Tigray, Afar, northern Amhara, and refugees from Eritrea.

### Study participants

Patients (aged>18 years) admitted to the surgical ward, who were taking antibiotic therapy or were candidates for antibiotic therapy/prophylaxis, were included in the study. Patients who had been admitted for less than 24 hours and refused to give consent were excluded. A sample of 222 patients was calculated using a single population proportion formula [30, 31] assuming a 76% proportion of antibiotic use related problems [16], 95% confidence level, 5% margin of error, and 5% contingency for nonresponse rate. From a total of 295 patients, 23 patients were excluded from the study due to the duration of hospitalization being less than 24 hours [13] and their unwillingness to provide consent [8].

### Data collection procedure

Patients were recruited during admission using a simple random sampling technique. All patients were followed up daily until discharge. Data from each patient were collected daily to check for any changes in the treatment. Written informed consent was obtained from all participants after a full explanation of the purpose of the study. The data were collected through patient interviews and expert reviews of patient’s medical, medication, and laboratory records. Fifth-year clinical pharmacy students were employed to collect data for this study. The data collectors were trained on the objectives of the study and methods of data collection.

### Antibiotic use related problems identification and assessment

Antibiotic use related problems are identified and classified using the Cipolle’s method [15] followed by a consensus meeting with a panel of experts including surgical specialists and clinical pharmacists. The appropriateness of antibiotics use was evaluated based on recent international guidelines including the Infectious Disease Society of America (IDSA), American Society of Health System Pharmacists (ASHP), and World Health Organization (WHO 2016) [32].

### Definition of terms

General practitioner is a medical graduate whose practice is not limited to specific class of medicine. A surgical resident is a doctor who has completed medical school and is in training for a surgical specialty. Surgical specialist, also known as surgeon, is a physician who is specially trained to perform medical surgery.

Antibiotic use related problem refers to any unwanted incident related to antibiotic therapy that actually or potentially affects the desired goals of treatment [15]. According to Cipolle’s method, antibiotic use related problems were categorized into seven major groups including unnecessary antibiotic therapy, the need for additional antibiotic therapy, ineffective antibiotic therapy, dosage too low, adverse drug reaction, dosage too high, and noncompliance.

The categories of antibiotic use related problems were defined and identified based on Cipolle’s method [15] as follows:

Unnecessary antibiotic therapy is considered when:

- There was no valid medical indication for the antibiotic therapy at this time,
- Multiple antibiotics were prescribed for an infection that required single antibiotic therapy
- Antibiotics were prescribed for non-bacterial infections including viral infections
- The medical condition could have been more appropriately treated with nondrug therapy

Need for additional antibiotic therapy was considered when:

- The medical condition required the initiation of antibiotic therapy
- Prophylactic antibiotic was required to reduce the risk of developing surgical site infection.
- The medical condition required additional antibiotic therapy to achieve synergistic or additive effects.

Ineffective antibiotic therapy was considered when:

- The least effective antibiotic was used while the most effective antibiotic was available
- The antibiotic was used for the treatment of infection caused by bacteria resistant to the antibiotic
- The antibiotic used was not an effective product for the bacterial infection being treated

Dosage too high was considered when:

- The dose of antibiotics was too high
- Dosing interval was too short
- The duration of antibiotic was too long
- A drug interaction that could result in a toxic blood concentration level of the antibiotic occurred
- The dose of the antibiotic was administered too rapidly

Dosage too low was considered when:

- The dose of the antibiotic was too low to produce the desired response
- A drug interaction that could reduce the bioavailability of the antibiotic occurred
- The duration of antibiotic was too short to produce the desired response
- The dosage interval was too long to produce the desired response

Adverse drug reaction was considered when:

- The antibiotic caused an undesirable reaction that was not dose-related
- The drug interaction caused an undesirable reaction that is not dose-related
- The antibiotic caused an allergic reaction
- The antibiotic was contraindicated due to risk factors
- The dosage regimen was administered or changed too rapidly

Non-adherence was considered if a patient failed to take medications appropriately due to one of the following reasons:

- Forgetfulness
- Lack of understanding of the instructions
- Unwillingness to take the medication
- Inability to swallow or self-administer the drug product appropriately
- Inability to afford
- Availability problem

### Data analysis

Data were entered into an EPI data management and analyzed using the Statistical Package for the Social Science (SPSS version 21.0). All statistical tests were performed using the SPSS software. We used descriptive statistics to summarize patients’ baseline characteristics as well as the prevalence and type of antibiotic use related problems. We checked multicollinearity among predictor variables using the variance inflation factor (VIF) and none were found to be collinear. Univariable logistic regression analysis was performed to determine the association of each independent variable with antibiotic use related problems. Moreover, independent variables with p <0.25 in univariable analysis were included in multivariable binary logistic regression model to identify predictors of antibiotic use related problems. Statistical significance was set at p <0.05.

### Ethical approval and informed consent

Approval for this study was obtained from the Ethics Review Committee of the School of Pharmacy, College of Health Sciences, Mekelle University. We fully explained the purpose of the study to each participant included in the study and written informed consent was obtained from each participant. The personal information was entirely confidential and protected. All methods were performed in accordance with the approved institutional guidelines.

## Results

### Socio-demographic related characteristics

A total of 272 patients were included in this study. Of these, 52.6% were males. The mean age was 44±17.90 years and the majority (59.2%) were urban dwellers. More than two-thirds (69.5%) were married and 33% had no formal education. In our study, 9.2%, 5.5%, and 4% were alcohol drinkers, smokers, and chat chewers, respectively. One-fourth of the participants used traditional medicine for their illnesses (Table 1).

**Table 1:** Sociodemographic characteristics among hospitalized patients at surgical ward of ACSH, 2018/2018, (n = 272).

Characteristics	Number (%)
Sex	
Male	143 (52.6)
Female	129 (47.4)
Age category in year	
< = 45	150 (55.1)
>45	122 (44.9)
Residence	
Urban	161 (59.2)
Rural	111 (40.8)
Marital status	
Single	69 (25.4)
Married	189 (69.5)
Widowed	6 (2.2)
Divorced	8 (2.9)
Educational status	
Illiterate	90 (33.1)
Primary school	74 (27.2)
Secondary school	45 (16.5)
Higher institute	63 (23.2)
Smoking status	
No	257 (94.5)
Yes	15 (5.5)
Chewing status	
No	261 (96)
Yes	11 (4)
Alcohol consumption	
No	247 (90.8)
Yes	25 (9.2)
Traditional medicine use	
No	204 (75)
Yes	68 (25)

### Diseases related characteristics

Nearly one-third (32%) of the participants had one or more comorbidities and 37.5% had been hospitalized for more than two weeks. More than one-third (38.6%) had a history of prior hospitalization and 31.3% had a history of anti-microbial use within the past three months (Table 2).

**Table 2:** Diseases related factors among hospitalized patients at surgical ward of ACSH, 2018/19, (n = 272).

Characteristics	Number (%)
Co-morbidity	
No	185 (68)
Yes	87 (32)
Duration of hospitalization in days	
< = 14 days	170 (62.5)
>14 days	102 (37.5)
History of hospitalization	
No	167 (61.4)
Yes	105 (38.6)
History of antimicrobial use within the past 3 months	
No	187 (68.8)
Yes	85 (31.3)

### Treatment related characteristics

The mean number of medications per patient was 4±1.26. Ceftriaxone (66.7%) was the most frequently prescribed drug, followed by metronidazole (30.5%) and cloxacillin (16.2%). Majority (57%) of the antibiotics were prescribed by surgical residents (Table 3).

**Table 3:** Treatment related factors among hospitalized patients at surgical ward of ACSH, 2018/19, (n = 272).

Characteristics	Number (%)
Number of medications per patient	
<5	184 (67.6)
≥5	88 (32.4)
Status of the prescriber	
General practitioner	47 (17.3)
Surgical resident	155 (57)
Surgical specialists	70 (25.7)
**Commonly used antibiotics**	
Ceftriaxone	182 (66.7)
Metronidazole	83 (30.5)
Cloxacillin	44 (16.2)
Amoxacilin	36 (13.2)
Cephalexin	32 (11.8)
Ciprofloxacin	32 (11.8)
Vancomycin	26 (9.6)
Ceftazidime	25 (9.5)
Gentamycin	20 (7.4)

### Antibiotic use related problems

A total of 235 antibiotics use related problems with a mean of 0.86± 0.82 problems per patient were identified. Nearly two-thirds (61.4%) of the participants experienced antibiotic use related problems. The commonly identified antibiotic use related problem were the need for additional drug therapy (29.4%), unnecessary drug therapy (15%), and dosage too high (12.1%) (Table 4).

**Table 4:** Types and causes of antimicrobial use related problems among surgical ward patients of ACSH, 2018/19, (n = 272).

Type of drug therapy problem	Reasons	Proportion with regard to patients (n = 272), n (%)	Proportion with regard to the total number of problems (n = 235), n (%)
Unnecessary drug Therapy	Duplicate therapy.	30 (11)	31 (13.2)
	No medical condition.	11 (4)	12 (5.1)
Needs additional drug therapy	Untreated indication	31 (11.4)	34 (14.5)
	Preventive or prophylactic	39 (14.3)	42 (17.9)
	To attain synergistic or additive effects.	10 (3.7)	11 (4.7)
Ineffective drug therapy	Not effective for condition	8 (3)	9 (3.8)
	More effective drug available.	14 (5.1)	14 (6)
Dosage too high	Duration too long	33 (12.1)	33 (14)
Dosage too low	Dose to low	11 (4)	11 (4.7)
	Dosage interval infrequent	4 (1.5%)	4 (1.7)
	duration of drug therapy too short	6 (4.6)	6 (2.6)
Adverse drug reaction	Allergy reaction	3 (1.1)	3 (1.3)
	Contraindication	3 (1.1)	3 (1.3)
Non-compliance	Forgetfulness	6 (2.2)	7 (3)
	Cost	8 (2.9)	9 (3.8)
	Availability	6 (2.2)	6 (2.6)

### Commonly involved class of antibiotics in drug related problems

**Fig 1:**
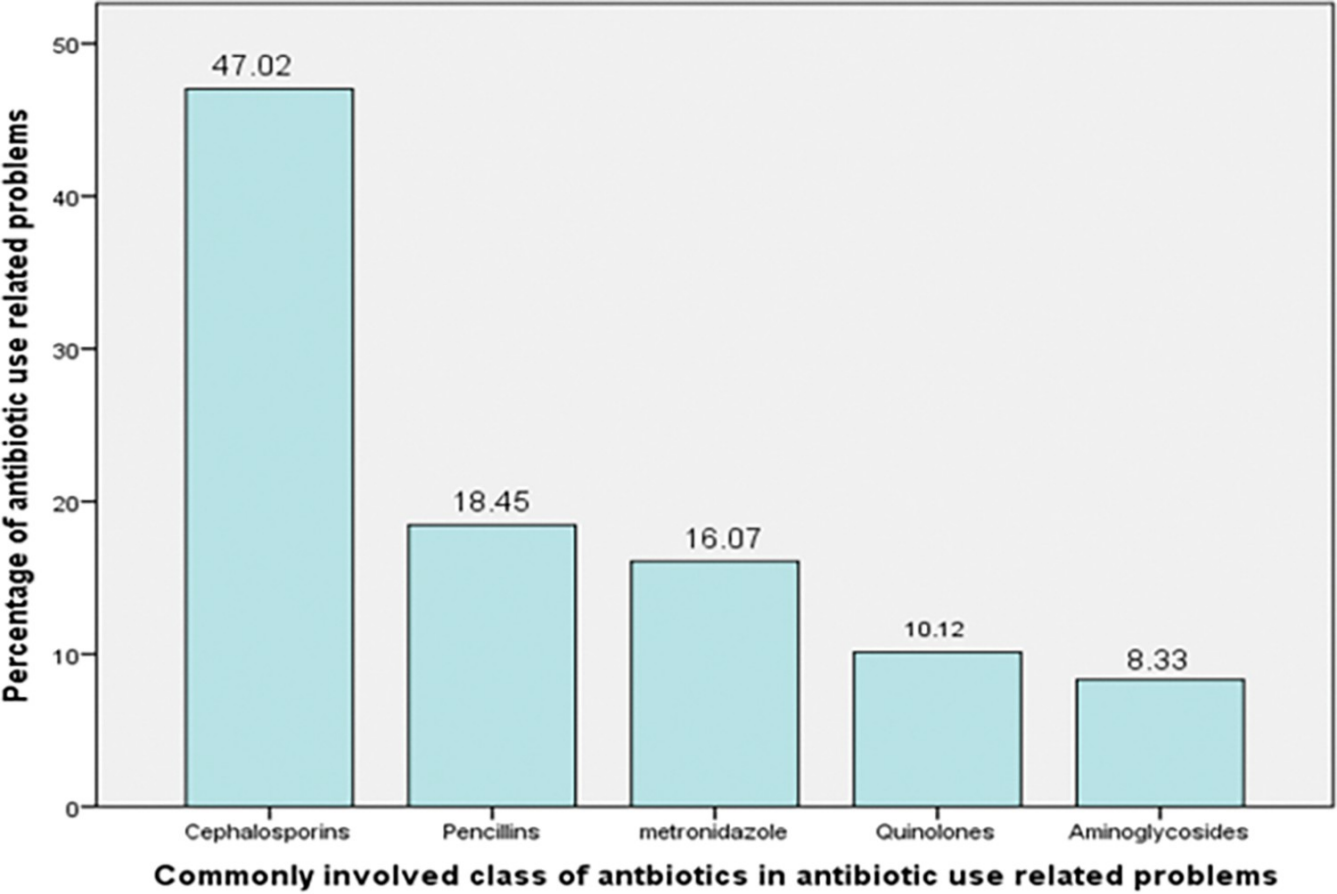
Commonly involved antibiotics in antibiotics use related problems among admitted surgical patients in ACSH, 2018/19, (n = 272).

Cephalosporins (47.02%) were the most commonly implicated class of antibiotics in drug related problems, followed by penicillins (18.45%) and metronidazole (16.02%) (Fig 1).

### Determinants of antibiotic use related problems

As illustrated in Table 5, variables with P<0.25 in the univariable analyses were included in the multivariable logistic regression model. The full model containing all variables was statistically significant (Chi-square = 66.993, df = 8, P<0.001). In the multivariable analysis, prolonged hospitalization (Adjusted odds ratio (AOR): 3.57, 95% confidence interval (CI):1.91–6.70) and number of medications ≥5 (AOR: 2.08, 95%CI: 1.10–3.94) were predictors of antibiotic use related problems. Moreover, patients whose medications were prescribed by general practitioners (AOR: 10.27, 95%CI: 4.13–25.58) and surgical residents (AOR: 2.28, 95%CI: 1.12–4.63) were more likely to experience antibiotic use related problems than those patients whose medication were prescribed by surgical specialists (Table5).

**Table 5:** Multivariate logistic regression analysis of factors associated with antibiotic use related problems at surgical ward of ACSH. 2018/2019, (n = 272).

Variables	DTP		COR (95% CI)	p-value	AOR (95%CI)	p-value
	No, n (%)	Yes, n(%)				
Sex						.
Male	60(57.1)	83(49.7)	1	1	1	1
Female	45(42.9)	84(50.3)	1.35(0.83–2.21)	0.232	1.17(0.67–2.06)	0.581
Traditional medicine use						
No	84(80)	120(71.9)	1	1	1	1
Yes	21(20)	47(28.1)	1.57(0.87–2.81)	0.133	1.61(0.83–3.13)	0.158
Duration of hospitalization						
≥14 days	83(79)	87(52.1)	1	1	1	1
>14 days	22(21.0)	80(47.9)	3.47(1.98–6.07)	P<0.001	3.57(1.91–6.70)	<0.001
History of antimicrobial use in the past 3 months						
No	77(73.3)	110(65.9)	1	1	1	1
Yes	28(26.7)	57(34.1)	1.43(0.83–2.44	0.197	1.41(0.76–2.62)	0.281
Number of medications						
<5	83(79)	101(60.5)	1	1	1	1
≥5	22(21)	66(39.5)	2.47(1.40–4.33)	0.002	2.08(1.103–3.94)	0.024
Co-morbidity						
No	79(75.2)	106(63.5)	1	1	1	1
Yes	26(24.8)	61(36.5)	1.75(1.02–3.01)	0.044	1.34(0.71–2.50)	0.365
Prescribers status			1	1	1	1
General practitioners	11(10.5)	62(37.1)	10.38(4.44–24.30)	<0.001	10.27(4.13–25.58)	<0.000
Surgical residents	59(56.2)	86(51.5)	2.69(1.40–5.14)	0.003	2.28(1.12–4.63)	0.023
Surgical specialists	35(33.3)	19(11.4)	1	1	1	1

Independent predictor at P < 0.25, COR, Crude odds ratio, AOR, adjusted odds ratio

## Discussion

Currently, antibiotic misuse has become a global crisis particularly in developing countries [21, 33]. Hence, identification and sharing of information regarding antibiotic use related problems and their determinants for hospital-based patients are fundamental to creating awareness among clinicians and developing preventive strategies for proper care of patients. Therefore, our study investigated the magnitude of antibiotic use related problems and their determinant factors among hospitalized surgical patients.

Appropriate use of antibiotics is crucial to prevent and control infection in surgical patients and is a key strategy to control antibacterial resistance [34, 35]. However, nearly two-thirds (61.4%) of the participants experienced at least one antibiotic use related problems in this study. In agreement with our study, similar findings were reported from surveys conducted in Pakistan [36], the United States of America [37], and Ethiopia [23]. In contrast, our findings were higher than those reported from Turkish (46.7%) and Ugandan (42%) studies [19, 38]. On the other hand, our finding was lower compared to the findings reported in Ghana and southwest Ethiopia [39], where antibiotic use related problems were identified in 86.6% and 80.1% of the participants, respectively. The possible reasons for these disparities might be due to the differences in population demographics, healthcare providers’ expertise and experiences, level of healthcare facilities, clinical characteristics, medication therapy used, and methods of antibiotic use related problems identification and classification.

In our study, the most frequent type of antibiotic use related problem was the need for additional antibiotic therapy (29.4%) followed by unnecessary drug therapy (15%) and dosage too high (12.1%). In line with our study, the need for additional drug therapy was the most frequent type of drug therapy problem in surveys conducted in southwest Ethiopia ranging from 29.6% to 31.3% [16, 39]. Moreover, unnecessary antibiotic therapy was the second most frequently encountered antibiotic use related problem in a similar study conducted in Jimma, Ethiopia [16]. An overdose of antibiotics was prescribed for 15.8% of patients in southwest Ethiopia [39] which is comparable with our findings.

Cephalosporins and penicillins were the drugs most commonly associated with antibiotic use related problems in the current study which was consistent with a study conducted in Pakistan [36]. This might be because these drugs were the most frequently prescribed drugs in our study and the Pakistan study [36]. The more the drug is used, the more likely it is to get involved in drug related problems [26]. In contrast to our study, tetracyclines and fluoroquinolones were the most frequently involved class of drugs in drug therapy problems in Ataye hospital, located in northeast Ethiopia [40]. This variance could be due to the difference in the study population in which our study was conducted in surgical inpatients while the study in Ataye hospital was carried out in outpatients.

In our study, prolonged hospitalization was a predictor of antibiotic use related problems which is consistent with other studies conducted in Ethiopia [16, 23]. This could be explained by the fact that the longer the hospital stay, the greater the total number of antibiotic exposures and the more the total number of drugs used, the more the risk of drug interaction, adverse drug events, and non-compliance. Another possible explanation is that the longer the hospital stays, the more the risk of developing hospital acquired infections which in turn results in extra costs that negatively affect the compliance of the patients to the prescribed drugs due to the affordability problem.

In the current study, patients with multiple medications (≥5 drugs) were two times more likely to develop antibiotic use related problems than patients with a smaller number of medications. Similar findings have also been reported from related studies conducted in Zambia [26] and Ethiopia [39]. The possible justification for this finding is that patients with multiple medications could be reluctant to take their medication appropriately owing to the inconvenience, safety, and cost issues [41]. Moreover, the higher the number of medications, the higher the risk of drug interaction which could affect the safety and/or efficacy of antibiotic therapy [41].

Patients whose medications were prescribed by general practitioners and surgical residents were more likely to experience antibiotic use related problems than those whose medications were prescribed by surgical specialists in our study. In agreement with our study, qualification/level of training of medical practitioners was a determinant of antibiotic use related problems in Uganda and Tanzania studies [19, 42]. This could be explained by the fact that healthcare providers with few years of training / less experience have higher rates of antibiotic prescription, regardless of whether they are indicated or not. However, healthcare providers with higher qualifications and those with better opportunities for updating knowledge have lower rates of antibiotic prescription as they are prescribed based on indication [43]. These findings indicated that there is a lower rate of irrational prescribing with higher qualifications and rational prescribing could be improved with continuing medical education to all prescribers. Therefore, surgical practitioners with higher qualification including surgical specialists need to be involved in patient evaluations and antibiotic prescribing.

Our study had some limitations. Since the findings of this study could be affected by the differences in population demographics, disease distribution, healthcare system, qualification of healthcare providers and methods employed, it should be extrapolated to other countries with caution. Moreover, this study was a single-center study; therefore, it might not be generalizable to the general population.

## Conclusion

Antibiotic use related problems were common among hospitalized surgical patients. Cephalosporins were the most commonly involved drugs in drug therapy problems followed by pencillins and metronidazole. Prolonged hospitalizations, number of medications, and qualification of practitioners were predictors of antibiotic use related problems. Therefore, more emphasis should be given topatients with prolonged hospitalization and multiple medications. Moreover, an integrated multidisciplinary team with higher qualifications including surgical specialists and clinical pharmacy specialists should be involved in the medication review and patient monitoring process in order to improve overall outcomes and reduce antibiotic use related problems and associated burdens in surgical patients.

## Supporting information

S1 Checklist STROBE statement—checklist of items that should be included in reports of *cross-sectional studies*. (DOCX)
